# Evaluation of Plant-Based Byproducts as Green Fining Agents for Precision Winemaking

**DOI:** 10.3390/molecules27051671

**Published:** 2022-03-03

**Authors:** Andrea Versari, Arianna Ricci, Annacarla Brioni, Cristian Galaz Torres, Carolina Alejandra Pavez Moreno, Javiera Concha García, Giuseppina Paola Parpinello

**Affiliations:** 1 Department of Agricultural and Food Sciences, University of Bologna, Piazza Goidanich 60, 47521 Cesena, Italy; andrea.versari@unibo.it (A.V.); annacarla.brioni@studio.unibo.it (A.B.); cristian.galaz2@unibo.it (C.G.T.); carolina.pavez2@unibo.it (C.A.P.M.); jaconcha2@uc.cl (J.C.G.); giusi.parpinello@unibo.it (G.P.P.); 2 CIRI-AGRO Inter—Departmental Centre for Agri-Food Industrial Research, Alma Mater Studiorum, University of Bologna, Via Q. Bucci 336, 47521 Cesena, Italy

**Keywords:** wine tannins, wine polyphenols, wine fining, patatin, surface response methodology, Freundlich adsorption isotherm

## Abstract

Consumers are increasingly looking for foods, including wine, that are free of animal-derived proteins. This study seeks to evaluate patatin, a new, plant-based and allergen-free fining agent, by comparing it with the fining agents polyvinipolypyrrolidone, bovine serum albumin, and methylcellulose. Specifically, its effects on the phenolic profile of enological tannins were analyzed with four spectrophotometric assays: OD 280 nm, Folin–Ciocâlteu, Adams–Harbertson, and methylcellulose. In addition, changes in the polyphenol composition of Sangiovese red wine were determined by UV-Vis spectrophotometry and HPLC with adsorption trials, and the solid–liquid interaction in a wine solution was modeled by both Langmuir and Freundlich equations. Our findings highlight the occurrence of systematic proportional error between the selected spectrophotometric assays. As a result, direct comparisons of protein precipitation assays can be made only among results obtained with the same spectrophotometric method. However, it is clear that patatin has an impact on the phenolic profile of Sangiovese red wine: it removes simple phenolics (gallic acid, (+)-catechin, (–)-epicatechin, epicatechin gallate, syringic acid, fertaric acid, coutaric acid, and rutin) as well as both oligomeric and polymeric tannins to different extents. In concentrations of less than 1 g/L, the patatin isotherm showed a linear relation between the equilibrium concentration and the quantity absorbed, obeying the Freundlich model reasonably well (K_F_ 1.46; 1/n 1.07; R^2^ 0.996 with 1/n > 1). Thus, the adsorption process is strongly dependent on the fining dosage.

## 1. Introduction

An increasing number of consumers demand traceability along the entire food production chain for several reasons, including to avoid the use of animal proteins as coadjuvants in plant-based products such as wine [1]. To achieve sustainable, organic, and environmentally friendly wines, enologists are seeking plant-based and allergen-free alternatives to traditional fining agents. Fining improves wine stability and enhances its characteristics while respecting its structure. A fining agent removes unwanted wine components by creating an almost insoluble protein–polyphenolic complex that precipitates from the solution. Indeed, complex formation has received extensive attention and the polyphenolic compounds involved in this complex have become the target for red wine treatments. Traditional fining agents include proteinaceous compounds such as casein, isinglass, albumin, and gelatin, which remove tannins and/or brown polymeric phenols; and polyamide materials such as polyvinylpolypyrrolidone (PVPP), which remove monomeric and small polymeric phenols [2]. Considering the allergenic and environmental drawbacks of some of the traditional fining agents, the search for an alternative is currently in progress. Potential substitutes include yeast protein extracts [3,4], grape pomace [5], cork powder [6], recycled PVPP [7], and vegetable proteins [8].

Unfortunately, fining agents are seldom perfectly selective. Valuable components may be partially removed from the wine, particularly when surplus fining agent is used (called overfining); its incomplete precipitation can not only cause wine instability (i.e., haze) but can also be a health concern if the agent has allergenic potential [9,10]. An in-depth evaluation of the effect of protein fining on the composition of wine shows that only a minor amount of the total phenolic content is removed, despite the lower astringency perception by sensory analysis [11]. According to Cheynier et al. [12], this apparent contradiction may be due to the combining of the remaining protein with the potentially astringent tannins to form a soluble or colloidal substance, so that the tannins are ‘unavailable’ to generate an astringent response. The evidence for the existence of soluble molecular assembly processes (“aggregates”), particularly involving tannins, proteins, and polysaccharides, has been thoroughly substantiated [13]. Ongoing concerns about residual proteins and related health issues have increased the use of fining agents made of plant proteins (e.g., cereals, grape seeds, potatoes, and legumes) as alternatives to potentially allergenic animal proteins (e.g., casein, gelatin, egg, and fish proteins) [14]. In fact, a new allergen-free plant protein (known as patatin) made from potatoes, the second-most wasted food ingredient in the world [15], has been recently approved at the EU level (Reg. EU 2019/924). Patatin does not require labeling as a potential allergen (unlike casein and egg white, for example) and is suitable for vegetarians and vegans as well (unlike gelatin, for example). The ability of patatin to reduce the flavanol compounds responsible for astringency in red wines was uncertain [8,16], as was the effect of patatin dosage on the time course of tannin and phenolic removal in red wine [17,18].

With the growing interest in patatin as a fining agent, there is a need to ascertain its adsorption of the phenolic compounds of red wines. The capacity of fining agents is best evaluated with a thorough understanding of adsorption processes in winemaking. Data from adsorption isotherms provide information on the affinity and capacity of the tested adsorbent [19]. Further, the isotherm can be fitted to both model wine solutions and real wines. Spectrophotometric methods, often used for routine analysis by industry, are valuable to researchers as well. The chemical reactivity and protein-binding capacity of the tannins are of great interest for quality control in winemaking; selecting an appropriate method may seem overwhelming to the nonspecialist, as there is no perfect method so far. In general, chemical assays are most useful for determining the amount of tannin in a sample, whereas protein-binding assays can be useful for evaluating the potential interaction of tannins in wine.

Accordingly, the aim of the study is (i) to compare selected spectrophotometric assays for the analysis of polyphenolic compounds and tannins; (ii) to characterize the polyphenolic–protein interactions, specifically with patatin, focusing on relevant implications concerning red wine fining and tannin assays.

## 2. Results

### 2.1. Analytical Methods Comparison

Four classic spectrophotometric assays for total phenolic content and tannin analysis were compared (Table 1A,B). The Adams–Harbertson assay (AH) represents a link between the Folin–Ciocalteu (FC) for total phenolic content and the methylcellulose precipitable (MCP) tannin assay. The sensitivity of the selected tannins was ascertained using the addition method, which showed an overall satisfactory correlation (Table 1). Although the four methods are closely correlated with each other (range R^2^: 0.779–0.999), each of the spectrophotometric assays yields different slopes between absorbance and tannin concentration due to the distinct origin and chemical composition of each tannin. The results of those assays must therefore be interpreted carefully to avoid misinterpretation of bias among methods due to interference from wine matrix. Tannins (i.e., polymeric units) are only a portion of the total phenolic compounds, which include small moieties as well, as the comparison between AH assays for total phenolics and tannins makes clear. In particular, AH and MCP precipitate tannins to different extents, most probably due to differences in selectivity towards the several types/subclasses of tannins.

**Figure 1:**
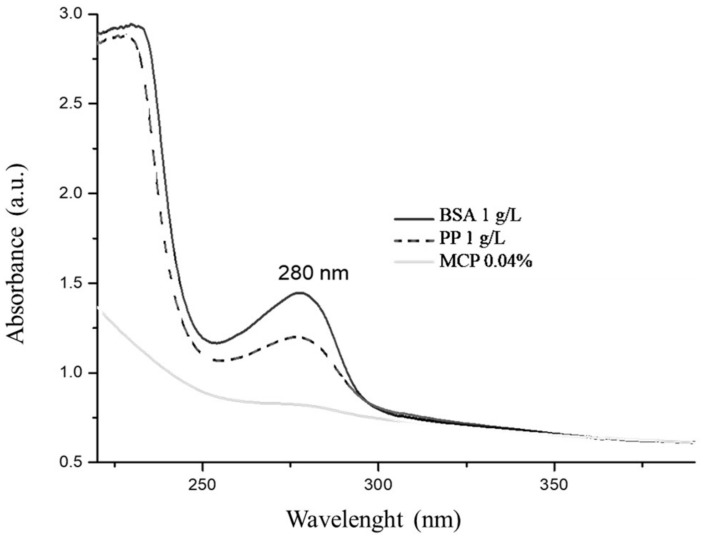
UV-Vis spectra of BSA (1 g/L), PP (1 g/L), and MCP (0.04%) in model wine solution. Legend: bovine serum albumin (BSA); patatin (PP); methylcellulose (MCP).

More insight into the preliminary results of the spectrophotometric assay comparison was obtained by considering the full UV spectrum of absorption values of selected adsorbent agents. For both BSA and PP, there was a high signal in the 220–400 nm range with a peak at 280 nm; the absorbance for MCP was negligible in comparison (Figure 1). The peak at 280 nm is a concern, because if residual fining agents occur at a detectable level they can interfere with the estimation of polyphenolic compounds in wines.

### 2.2. Fining Trials in Model Wine Solution

The current study tested three adsorbent agents—BSA, PVPP, and PP—to better understand their capacity to adsorb phenolic compounds in red wine, including enological tannins. The effects of BSA and PVPP on phenolic compounds was initially evaluated for four enological tannins in model wine solution: grape skin proanthocyanidins (SKN) and grape seed procyanidins (PIP) as condensed tannin, and gallotannins (GAL) and ellagic tannin (ELL) as hydrolysable references. Several RSM experimental trials were designed in order to develop an empirical model relating the responses to the control variables (see Supplementary Materials, Figures S1 and S2).

RSM showed that maximum quantification is achieved using different tannin/precipitant ratios. In particular, compared with the A-H assay, BSA slightly higher than 0.2 g/L would be able to quantify almost all the tannin contained in a 1 g/L model wine solution, with maximum effectiveness at a BSA concentration of 2 g/L (Figure S1). Excessive doses of BSA (>4 g/L) would result in a slight reduction in the quantification of any tannins analyzed. In contrast, PVPP exhibited a different behavior: the maximum quantification of tannins at 1 g/L was achieved with precipitant doses higher than 2 g/L (Figure S2). Excessive dosage (up to 4 g/L) led to a reduction in tannin precipitant capacity. Based on these early findings, we can conclude that the optimum tannin powder/PVPP ratio for maximum precipitation (and thus, quantification) is 1:2.5, whereas in the case of BSA, the optimal ratio is 1:2.

**Figure 2:**
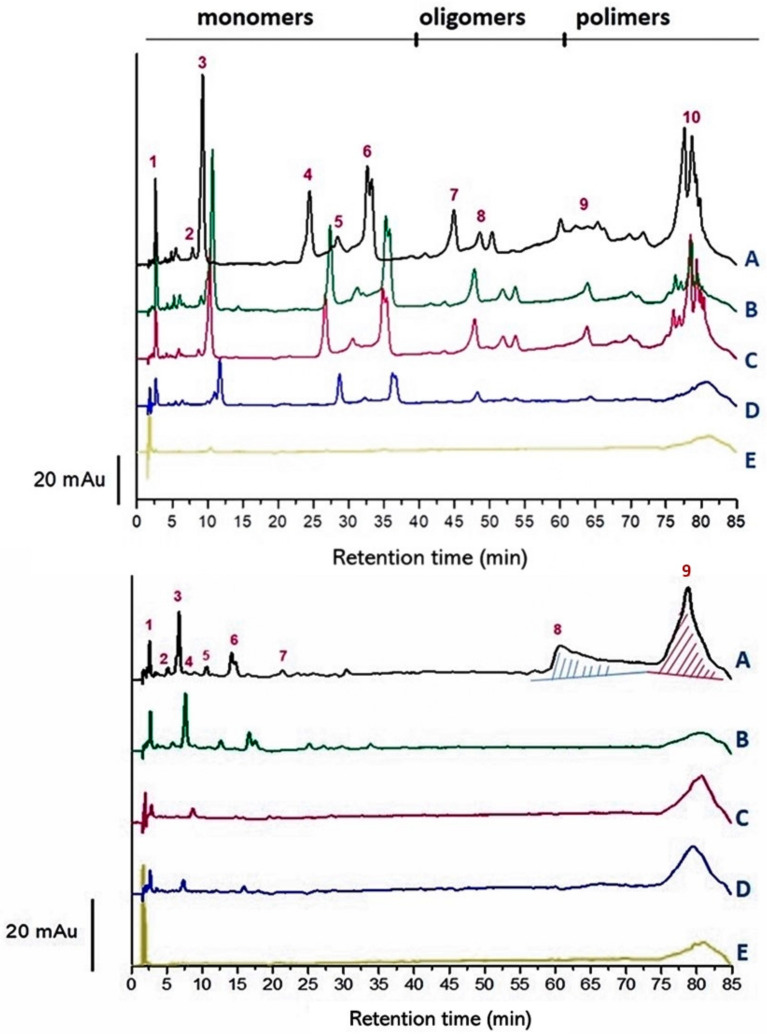
HPLC profile at 280 nm of fining trials in model wine solutions with gallotannin (**top**) and grape seed proanthocyanidin (**bottom**) at 0.6 g/L. Legend of fining agents: (A) control; (B) patatin (PP) at 1 g/L; (C) BSA at 1 g/L; (D) PVPP at 1 g/L; (E) MCP at 0.04%.

Following RSM screening, further (un)targeted HPLC analyses were run on Sangiovese red wine to highlight the adsorbents’ selectivity after the addition of chosen adsorbent materials. The HPLC profile at 280 nm clearly showed the presence of several peaks along the entire time course of analysis, most of them well-resolved (Figure 2).

Three main regions were identified (based on previous findings): monomeric (i.e., gallic acid, catechin, epicatechin, and syringic acid), oligomeric, and polymeric polyphenolic compounds [20]. The fining agents clearly removed the targeted polyphenolic compounds differently, with MCP being by far the most active over the entire HPLC profile, removing from monomers to polymers hump. The effect of PP was comparable with PVPP, especially when considering gallotannins; the former proved to be effective at removing polymeric compounds as well. The rare peak values higher than 100% are negligible, considering the precision of the method (about 5%) and the very small area under the peak involved: (ca. 0.7% of the total peak area) for catechin (peak n. 2) in gallotannin trials and flavanol-3 (peak n. 5) in grape seed tannin trials (Table 2).

### 2.3. Fining Trials on Sangiovese Red Wine

**Figure 3:**
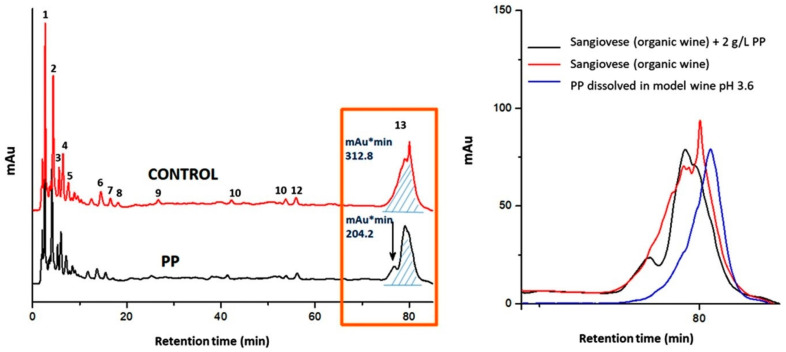
HPLC profile at 280 nm of Sangiovese red wine (control: top) and with PP fining agent at 2 g/L (bottom), with highlight on the hump of polymeric phenolic eluting at approx. 80 min (box on the right side).

Based on the preliminary findings using model wine solution, the dosage of PP was increased to 2 g/L in the trials using Sangiovese red wine. As expected, Sangiovese red wine showed an HPLC profile with many peaks at 280 nm: the early retention times correspond primarily to monomeric phenolic compounds and the late times primarily to polymeric phenolic compounds (Figure 3, Table 3). Although PP was able to remove about 35% of the latter (Table 3: peak n. 13), the chromatogram overlay of Sangiovese red wine with PP at approximately 80 min showed the presence of an extra peak which lacking in the control. The occurrence of a soluble tannin–protein complex was postulated to explain this finding. Indeed, the HPLC signal of PP alone overlapped those of polymeric phenolics at approximately 80 min. This result, consistent with the UV-Vis spectra properties of PP (Figure 1), will be exploited in future studies to obtain more insight into the tannin–protein interaction.

### 2.4. Measurement of Adsorption Isotherms of Tannins

**Figure 4:**
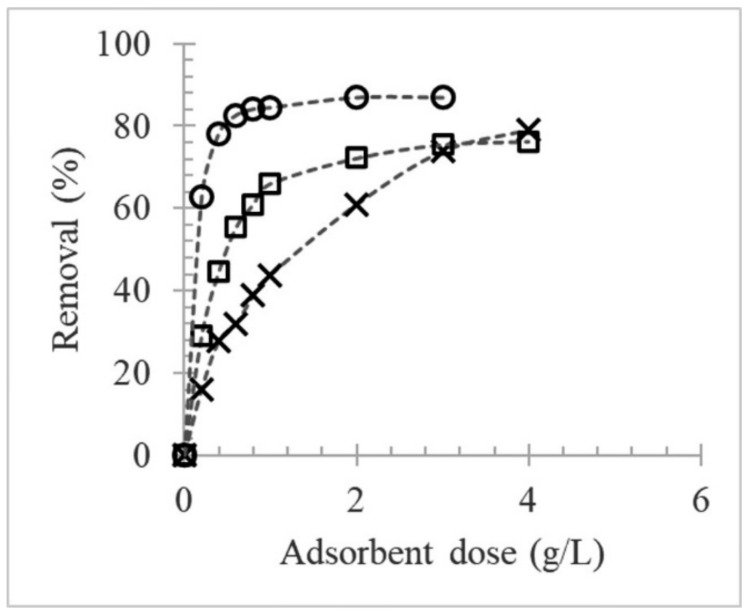
Effect of fining agent dose on removal of polyphenolic compounds from model wine solution with grape skin tannin (0.6 g/L). Legend: PVPP (X); PP (□); BSA (○).

Analysis of adsorption isotherms, which illustrate the whole adsorption process, can facilitate better control of the fining agents, thus helping achieve the desired result. The effect of adsorbent dose on the removal of polyphenolic compounds was investigated in model wine solution containing added grape skin tannin (0.6 g/L) by varying the fining agent loading from 0.2 to 4 g/L. Figure 4 shows that removal efficiency was related to the dose: the percentage removed rapidly increased with increasing adsorbent concentration until a maximum was approached, after which the percentage removed increased relatively slowly, until further dose increases had no effect. The fining agents removed the phenolic compounds up to 87% for BSA, followed by PVPP (79%) and PP (76%). This behavior could be due to the high availability and accessibility of adsorption sites or surface area at high doses.

**Figure 5:**
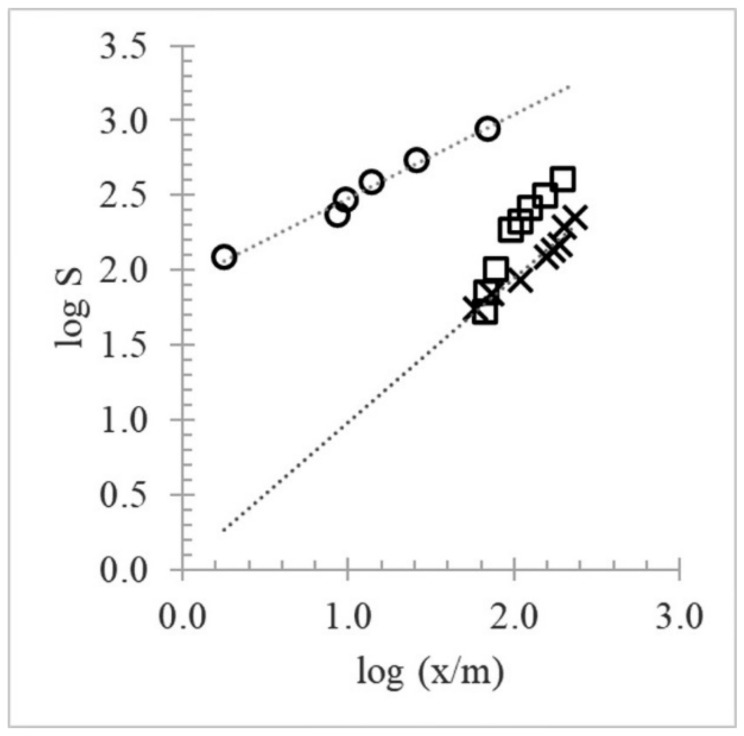
Freundlich plot of grape seed tannin adsorption by fining agents. Legend: PVPP (X); PP (□); BSA (○).

Furthermore, the experimental data for the tannin–fining agent interaction were investigated by comparing two well-known models, the Freundlich and Langmuir adsorption isotherms. Under our experimental conditions, the molar concentrations of the fining agents were unknown; therefore, the binding constants are only estimates of the true constants. Of the two models, the empirical Freundlich multisite adsorption isotherm showed the best linear fitting for BSA (K_F_ 84.06; 1/n 0.56; R^2^ 0.984) and PVPP (K_F_ 1.07; 1/n 0.96; R^2^ 0.958) (Figure 5), with high correlation coefficients and a value of 1/n < 1.

The process is considered viable, because the amount adsorbed decreases with increasing concentration. It is well-known that the Freundlich model assumes a heterogeneous multilayer adsorption surface and considers the sites having varying adsorption energies at different sites; thus, they are not equally available. However, unlike the interactions with BSA and PVPP, the tannin–PP isotherm did not comply with the Freundlich model at concentrations greater than 1 g/L. It was instead nonlinear with a negative curvature. These characteristics are tentatively attributed to (i) the high concentrations of PP with relatively high exposed hydrophobicity and/or (ii) the presence of a soluble tannin–PP complex, which interferes at 280 nm when the PP concentration is high (>1 g/L). Below 1 g/L, however, the PP isotherm showed a linear relation between the equilibrium concentration and the quantity absorbed that obeys the Freundlich model reasonably well. The corresponding adsorption parameters (K_F_ 1.46; 1/n 1.07; R^2^ 0.996) with 1/n > 1 indicate that the adsorption process is strongly dependent on the concentration. This dependence is characteristic of rather weak adsorption of the solute by the solid and strong lateral interactions between adsorbed molecules themselves once they become sufficiently concentrated on the surface [21].

## 3. Discussion

### 3.1. Analytical Methods Comparison

The correct interpretation of data from chemical assays for tannin requires an understanding of the specificity of each assay. Excess protein in the reaction mixture decreases the amount of precipitable complex that forms, resulting in an apparent decrease in the amount of tannin present [22]. In particular, the MCP assay creates a mixture that forms insoluble polymer tannin complexes, which then precipitate [23]. The method seems to be selective for grape tannins only, i.e., polymeric proanthocyanidins; it does not remove simple phenolics from the grape homogenate extracts [24]. Unlike in the two latter cited reports, our HPLC chromatograms from the fining trials on wine model solution (with enological tannins added) clearly show that MCP removed almost every phenolic compound from the treated samples (Figure 2). Furthermore, Mercurio and Smith [24] revealed that regression analyses of the MCP and AH tannin assays have slopes of 0.36 and 0.32 for grape and wine samples, respectively, revealing an almost three-fold difference in tannin concentration. Thus, the MCP assay reports three times the tannin content of the AH assay, which is based on BSA precipitation (demonstrated not to bind dimers), and the amount of tannin precipitated by BSA increased with the addition of every subunit from trimers to octamers [25].

Our findings provide a reasonable explanation for the systematic, proportional differences between the two methods mentioned. The reaction between tannin and protein is complex; thus, the results of protein precipitation assays can be compared only for results obtained under the same analytical conditions. Moreover, the choice of a suitable single standard, either tannin or protein, is critical for obtaining consistent and comparable results when analyzing tannins.

### 3.2. Fining Trials and Adsorption Isotherms

Previous studies on the effect of fining agents on red wine have focused on the phenolic composition, instead of modeling the activity as a function of the type and concentration of fining agent. The present findings can be used to tailor winemaking practices and improve analytical quality control. The amount of solute removed by a fining agent depends on the solute/agent adsorption equilibrium, which approaches a steady state as the wine’s solute concentration is reduced; therefore, the fining agent becomes increasingly less effective. From a practical point of view, the solute concentration is merely decreased to a point below a solubility condition (in a stability test) or a taste threshold (in a sensory test) [2]. Thus, the adsorption isotherms can help determine the most appropriate fining agent as well as predicting the performance of adsorption systems. According to Waterhouse et al. [9], tannin fining with gelatin follows the Freundlich model, where addition of tannin results in a decreasing fraction of tannin adsorbed, but without leveling off in the amount of tannin bound to the protein. Indeed, binding also depends on the isoelectric point (pI) of protein and is, thus, at a maximum (for most proteins) when the pH is at or near its pI value. Since patatin and BSA have similar isoelectric points of 4.6 and 4.7, respectively [26,27], this variable can be neglected in the present study.

In agreement with our findings, the adsorption isotherm of (−)-epigallocatechin gallate (EGCG) on BSA, which appears to be dominated by nonspecific hydrophobic interactions, is better described by the Freundlich model than the Langmuir model [28]. Further comparison on mass balance should consider the pH effect, as BSA undergoes conformational isomerization with decreasing pH, EGCG adsorption increases below pH 4.0 (fast “F” form: 40 × 129 Å) and increases even more for pH close to 3.0 (expanded “E” form: 21 × 250 Å) [29]. The Freundlich and Langmuir models are both suitable for modeling the adsorption of catechins onto PVPP at a neutral pH. The reaction was driven by the PVPP and catechin concentrations, and favored by low temperature, i.e., 20 vs 40 °C [30].

In a preliminary study on distilled water, the Freundlich equation showed that the adsorption of the hydroxybenzoic acids by PVPP is not selective [31]. Further experiments on model beer solution (5.0% ethanol at pH 4.0) revealed that PVPP preferentially removed the more highly hydroxylated phenolic acids, but similar selectivity was not found for the adsorption of dimeric flavanols. The Freundlich constants (K_F_, 1/n) for the adsorption of phenolic acids and beer flavanols by PVPP ranged between 0.02 and 10 for K_F_ and between 0.60 and 0.91 for 1/n [32]. These values are consistent with our findings on PVPP (K_F_ 1.07; 1/n 0.96). Mitchell et al. [33] showed that the trend of beer proanthocyanidins to bind to PVPP increased with the degree of polymerization [(*n* = 3) > (*n* = 2) > (*n* =1)] [33]. This trend was also confirmed in rosé wines, in which about 64% of the total flavanol content was removed by PVPP, with trimers slightly more adsorbed than dimers (79% vs. 72%) and much more adsorbed than monomers (43%) [34]. Interestingly, in model wine solution, the compounds having more affinity for PVPP were quercetin (100%) and catechin (67%) [35]; also, quercetin aglycone above 3 mg/L may also become insoluble [36]. On the contrary, BSA does not precipitate flavanol monomers and dimers, whereas the ability of BSA to precipitate condensed tannins increased with increased degree of polymerization from trimers (1000 μg) to octamers (50 μg) [25].

### 3.3. Practical Implication on Red Wine Properties

Wine stability represents one of the more debated topics in enology. Practically speaking, there is no ideal fining product, as all of them have specific selectivities that are more or less effective on each parameter, depending on the type of wine. It is thus essential to follow a sound scientific approach supported by an empirical model in order to select the most suitable product and the optimal dosage. For example, Aglianico red wine treated with patatin at 30 gr/Hl showed a significant decrease in astringency, due to the removal of polyphenols reactive toward salivary proteins [16]. Although potato-derived proteins (max dosage 25 gr/Hl) effectively reduced large proanthocyanidins in Montepulciano, Primitivo, and Nebbiolo red wines, the monomeric and oligomeric forms of flavanols, evaluated with vanillin assay, occasionally increased up to 4% compared with untreated wine [8]. This increase is consistent with our findings.

The occurrence of a soluble protein–tannin complex should be considered in winemaking—not just because of the technical interference among different analytical methods, but also because of the regulatory implications and the complex’s effect on wine’s sensory properties and stability. The International Organization of Vine and Wine [37] has recommended a limit of <0.25 mg/L for potentially allergenic residues of fining agent proteins in wine, above which a mention becomes compulsory on the wine label. Potato protein fining is significantly influenced by wine pH, ethanol concentration, and fining temperature [17]. The combined use of several fining agents is also a concern, since it can lead to synergistic or antagonistic effects. The complexity of the fining process and the large number of possible combinations make it difficult to predict the outcome [38].

## 4. Materials and Methods

### 4.1. Wines

Sangiovese red wine was obtained from grape clone FEDIT 30 ESAVE from a vineyard of approx. 2 ha located in Tebano (Ravenna, Italy; 44°17′24.9″ N; 11°47′08.6″ E) managed according to organic farming regulation (Reg. EC 834/2007), processed as previously described [39]. The wine has the following general composition: alcohol 13.0% *v/v*, pH 3.60, total acidity 4.50 g/L (the phenolic composition was analyzed as later described). Sangiovese is the main Italian red grape cultivar, with approx. 54,000 ha across the country; its origins, while not determined with certainty, are mainly considered to be in Romagna and Tuscany.

### 4.2. Chemicals and Fining Agents

The following chemicals and reagents were from commercial source: acetonitrile and formic acid for HPLC eluents were from Merck (Darmstadt, Germany); gallic acid, (+)-catechin, (–)-epicatechin, caffeic acid, syringic acid, and p-coumaric acid used for HPLC determinations and spectrophotometric calibrations, along with the Folin–Ciocalteu reagent, iron(III) chloride, and sodium dodecyl sulfate used for the spectrophotometric assays were from Sigma (Sigma–Aldrich, Milano, Italy).

Three fining agents were compared: (i) bovine serum albumin (BSA), a well-characterized model globular protein; (ii) potato protein called ‘patatin’ (PP), a nonallergenic protein; (iii) polyvinylpolypyrrolidone (PVPP), a resinous polymer that acts analogously to proteins in binding tannins. Moreover, the polysaccharide polymer methylcellulose (MCP), commonly used in enology laboratory assays to precipitate tannins, was also tested for comparison. BSA and MCP were purchased from Sigma (Sigma–Aldrich, Milano, Italy); PP and PVPP oenological additives were kindly supplied by Laffort (Italia, Alessandria, Italy).

### 4.3. Enological Tannins

Four enological tannins in the form of commercially available phenolic compounds were selected: two condensed tannins—one extracted from grape skins (SKN) and one from seeds (PIP); two hydrolysable tannins—one extracted from gallnuts (GAL) and one from ellagic acid (ELL). All extracts were kindly supplied by Laffort (Alessandria, Italy). Further information on the selected tannins, including their antioxidant capacity, is available in the literature [40].

### 4.4. Fining Trials

The affinity of the selected fining agents for phenolic compounds was investigated over a wide range of concentrations, in both model wine solutions and Sangiovese red wine. Stock solutions of dry tannins were prepared at 1 g/L in the model wine solution and further diluted for trial purposes (see Section 4.7). The model wine solution was made of 12.0% *v/v* ethanol (>99% purity) in distilled water HPLC-grade (Merck, Darmstadt, Germany), with the addition of L-tartaric acid 0.033 M and NaOH to reach pH 3.60. In the fining trials, both the fining agents and the enological tannins were tested at concentrations suitable for the isotherm adsorption test.

### 4.5. Spectrophotometric Determinations

The total polyphenol content (TPC) was analyzed with two methods. In the first, the optical density (OD) was measured at 280 nm [41] using a Cary60 UV–Vis (Agilent Technologies, Santa Clara, CA, USA), building a calibration curve with gallic acid as the reference standard and the following performances in terms of linearity (coefficient of determination R^2^ > 0.997; lack-of-fit test *p* > 0.05), precision (RSD < 2.8%), and limits of detection and quantification (0.5 and 2 mg L^−1^ gallic acid, respectively). The second was the Folin–Ciocâlteu (FC) method [42], using gallic acid as a reference standard normalized to 10 mm optical path; the FC method showed satisfactory performances in terms of linearity (coefficient of determination R^2^ > 0.998; lack-of-fit test *p* > 0.05), precision (RSD < 1.7%), and limits of detection and quantification (0.5 and 1.5 mg L^−1^ gallic acid). The wine tannins were measured by means of two well-known tannin-precipitation methods: the Adams–Harbertson (AH) protein precipitation tannin assay using BSA [43], with standard curve characterized by linearity (coefficient of determination R^2^ > 0.999; lack-of-fit test *p* > 0.05), precision (RSD < 4.0%), and limits of detection and quantification (8 and 25 mg L^−1^ catechin, respectively); the methylcellulose precipitable (MCP) tannin assay, based on MCP–tannin interactions, which form insoluble polymer tannin complexes (which precipitate). The latter assay is completed by subtracting the absorbance values at 280 nm of solutions both with and without precipitation [44], and the MCP method is performed as follows: linearity (coefficient of determination R^2^ > 0.999; lack-of-fit test *p* > 0.05), precision (RSD < 2.4%), and limits of detection and quantification (8 and 25 mg L^−1^ catechin, respectively). The two assays were selected for their different approaches to the quantification of pigmented polymers when testing for total tannin.

### 4.6. HPLC Analysis

Samples from fining trials were analyzed by a High-Performance Liquid Chromatography (HPLC) system equipped with a temperature control oven, photodiode array detector (DAD), and Chromeleon chromatography manager software v. 6.60 SP2 (Dionex DX500, Milano, Italy) was used for identification and quantification of polyphenolic compounds. All samples were filtered using 0.20 μm cellulose acetate membranes (Millipore, Milano, Italy) and directly analyzed with an Aquapore ODS-300 RP-C18 column (250 × 4.6 mm; 7 µm particle size; 300 Å porosity; Applied Biosystems, San Jose, CA, USA). Flava-3-ol compounds including (+)-catechin, (–)-epicatechin, and their derivatives, together with benzoic acids, i.e., gallic and syringic acids were quantified at 280 nm, whereas p-coumaric acid was at 308 nm and caftaric, caffeic, coutaric, and fertaric acids at 324 nm. Details on the HPLC method, including temperature conditions, eluents, and elution gradient have been extensively described in a previous work [20].

### 4.7. Measurement of Adsorption Isotherms of Tannin

The study of solid–liquid adsorption systems is commonly modeled using the Langmuir and Freundlich equations. In this study, the adsorption isotherms resulting from these equations describe the relationships between the equilibrium concentration of grape skin tannin in the adsorbent and liquid phases (model wine) by varying the adsorbent/tannin ratios. According to Blade and Boulton [45], the Langmuir (1) and the Freundlich (2) adsorption isotherms may be written as:

x/m = (x/m)_max_ × {[S]/[K_L_ + [S]]} (1)

x/m = K_F_ × [S]1/n (2)

where x is the amount of solute adsorbed on a mass m of solid at equilibrium time (mg/g) and S is the equilibrium solute concentration that remains in solution after adsorption (mg/L). The constants K_L_ and (x/m)_max_ (or K_F_ and 1/n) represent the binding constant related to the absorption pair; they can be calculated by plotting double reciprocal plots (x/m against 1/S) and a log–log plot (x/m against S) for the Langmuir and Freundlich equations, respectively, using regression analysis (Addinsoft XLSTAT 2018, Paris, France).

### 4.8. Statistical Analysis

Response Surface Methodology (RSM) is a statistical technique for optimizing the experimental conditions from a minimal number of experiments. It also gives enough information to estimate results for the purpose of designing a process [46]. This technique allowed us to investigate the relationship between the response (tannin residual content) and control variables (type of enological tannin, type of protein, and tannin–protein ratio) in order to predict the response values for a specific range of control variables. In this study, a Central Composite Design with three central point replicates to evaluate the curvature was applied using UNSCRUMBLER software (v. 9.7, Camo, Norway). The experiment consisted of 13 analyses for each precipitant and each tannin, with tannin concentrations ranging from 0 to 1 g/L and precipitant concentrations ranging from 0 to 5 g/L. Regression analysis (R^2^: coefficient of determination), the lack-of-fit test (α = 0.05), and one-way ANOVA were performed using XLSTAT software (v. 2018, Addinsoft, Paris, France).

## 5. Conclusions

Fining binds with targeted compounds and precipitates them, removing undesired components to improve wine quality (and conform to consumer preferences). Developing environmentally friendly fining agents is crucial for reducing wine loss and minimizing waste in the winemaking processes. Laboratory trials play a vital role in ensuring that the fining agent has the desired effect. Moreover, precision fining helps reduce the risk of wine oxidation by lowering the concentration of oxidized/oxidizable phenolic compounds. This study is the first to investigate the adsorption of patatin on phenolic components of red wine through equilibrium experiments and the Freundlich isotherm model. Sangiovese red wine and enological tannins contain simple, oligomeric, and polymeric flavanols. Adsorption trials in model wine solutions demonstrated that tannin concentrations dropped by fining agent in a dose-dependent way and that the fining performances were driven by the specific phenolic profiles of the matrices. These findings indicate that simple phenolics and tannin can be removed to different extents by fine-tuning the patatin concentration.

## Figures and Tables

**Table 1:** Calibration of total phenolics (1A) and tannins (1B) with UV-vis assays, lack of fit, and ANOVA results. Legend: Optical density 280 nm (OD 280 nm); Folin–Ciocâlteu (FC); Adams–Harbertson (AH); Methylcellulose (MCP); grape skin (SKN); grape seed (PIP); gallotannin (GAL); ellagic tannin (ELL); R^2^, coefficient of determination.

1A. Total Phenolics	OD 280 nm (g/L GAE)				FC (g/L GAE)				AH (g/L CE)			
Tannin Powder (g/L)	SKN	PIP	GAL	ELL	SKN	PIP	GAL	ELL	SKN	PIP	GAL	ELL
0.1	0.00 _c_	0.00 _C_	0.01 ^c^	0.01 ^C^	0.07 _a_	0.05 _A_	0.09 ^b^	0.06 ^A^	0.03 _b_	0.03 _B_	0.10 ^a^	0.02 ^B^
0.2	0.01 _c_	0.01 _C_	0.03 ^c^	0.01 ^C^	0.11 _a_	0.12 _A_	0.16 ^b^	0.10 ^A^	0.07 _b_	0.07 _B_	0.27 ^a^	0.06 ^B^
0.4	0.01 _c_	0.01 _C_	0.6 ^b^	0.02 ^C^	0.19 _a_	0.20 _A_	0.28 ^c^	0.18 ^A^	0.16 _b_	0.18 _B_	0.62 ^a^	0.16 ^B^
0.6	0.02 _c_	0.02 _C_	0.83 ^b^	0.02 ^C^	0.23 _b_	0.29 _B_	0.38 ^c^	0.22 ^B^	0.25 _a_	0.30 _A_	0.93 ^a^	0.30 ^A^
0.8	0.03 _c_	0.02 _C_	1.09 ^b^	0.34 ^B^	0.33 _b_	0.36 _B_	0.52 ^c^	0.30 ^C^	0.39 _a_	0.42 _A_	1.28 ^a^	0.43 ^A^
1.0	0.03 _c_	0.03 _C_	1.34 ^b^	0.43 ^B^	0.38 _b_	0.42 _B_	0.60 ^c^	0.37 ^C^	0.50 _a_	0.55 _A_	1.62 ^a^	0.51 ^A^
R^2^	1.000	0.994	0.974	0.762	0.988	0.985	0.996	0.996	0.994	0.997	1.000	0.992
Slope	0.031	0.029	1.549	0.492	0.344	0.407	0.570	0.337	0.532	0.582	1.685	0.574
**1B. Tannins**	**AH (g/L CE)**				**MCP (g/L CE)**							
**Tannin Powder (g/L)**	**SKN**	**PIP**	**GAL**	**ELL**	**SKN**	**PIP**	**GAL**	**ELL**				
0.1	0.00 _b_	0.00 _B_	0.00 ^b^	0.00 ^A^	0.05 _a_	0.00 _A_	0.11 ^a^	−0.01 ^A^				
0.2	0.05 _a_	0.06 _A_	0.00 ^b^	0.00 ^B^	0.00 _b_	−0.10 _B_	0.29 ^a^	0.05 ^A^				
0.4	0.15 _a_	0.18 _A_	0.31 ^b^	0.08 ^B^	0.13 _b_	0.08 _B_	0.82 ^a^	0.15 ^A^				
0.6	0.25 _b_	0.28 _A_	0.60 ^b^	0.22 ^A^	0.28 _a_	0.20 _B_	1.10 ^a^	0.18 ^B^				
0.8	0.35 _b_	0.37 _A_	0.86 ^b^	0.33 ^A^	0.44 _a_	0.21 _B_	1.50 ^a^	0.30 ^B^				
1.0	0.46 _b_	0.50 _A_	1.10 ^b^	0.39 ^B^	0.57 _a_	0.25 _B_	1.70 ^a^	0.55 ^A^				
R^2^	0.999	0.999	0.984	0.978	0.969	0.937	0.993	0.948				
Slope	0.509	0.544	1.296	0.477	0.638	0.351	1.893	0.559				

Legend: Letters represent the results of Tukey’s post hoc comparison tests (polyphenols) and *t*-test (tannins). For row values with different letters, samples have significantly different means (α = 0.05). SKN—lowercase subscript; PIP—capital subscript; GAL—lowercase superscript; ELL—capital superscript.

**Table 2:** Effect of fining agent on adsorption of phenolic compounds (%) from model wine solutions with gallotannin (top) and grape seed proanthocyanidin (bottom) at 0.6 g/L. For each fining agent, values of HPLC peak area are normalized to percentage compared with control (100%). See Figure 2 for peak numbering. Legend: Patatin (PP); Bovine Serum Albumin (BSA); Polyvinylpolypyrrolidone (PVPP); Methylcellulose (MCP).

Peak n.	Compound	PP (%)	BSA (%)	PVPP (%)	MCP (%)
1	gallic acid	99.5	31.8	75.4	nd
2	(+)-catechin	100.0	16.0	70.9	nd
3	epicatechin gallate	100.0	22.7	60.8	1.9
4	syringic acid	92.7	28.2	68.0	nd
5	oligomers gallic acid	84.6	22.6	63.8	nd
6	oligomers gallic acid	68.5	13.0	69.9	nd
7	oligomers gallic acid	62.1	10.2	63.9	nd
8	oligomers gallic acid	82.5	13.6	100.6	nd
9	polymers hump 1	43.7	nd	48.9	nd
10	polymers hump 2	46.6	27.2	89.1	nd
1	gallic acid	100.0	46.4	67.4	nd
2	(+)-catechin	91.3	nd	nd	nd
3	flavanol 1	94.2	18.4	19.9	nd
4	flavanol 2	39.1	nd	nd	nd
5	flavanol 3	100.0	nd	nd	nd
6	flavanol 4	80.2	nd	15.9	nd
7	(–)-epicatechin	100.0	nd	nd	nd
8	polymers hump 1	Nd	100.0	nd	nd
9	polymers hump 2	27.6	nd	79.4	31.0

**Table 3:** Effect of the PP fining agent (concentration 2 g/L) on adsorption of phenolic compounds (%) from Sangiovese organic red wine. Values of HPLC peak area are normalized to percentage compared with control (100%). See Figure 3 for peak numbering.

Peak n.	Compound	PP (%)
1	gallic acid	85.7
2	caftaric acid	83.8
3	fertaric acid	93.4
4	coutaric acid	82.5
5	(+)-catechin	95.2
6	epicatechin gallate	93.9
7	syringic acid	64.9
8	(–)-epicatechin	95.7
9	flavanol	65.3
10	flavonol glucoside-1	80.0
11	flavonol glucoside-2	56.8
12	rutin	92.8
13	polymers hump	65.3

## Data Availability

Not applicable.

## Supplementary Materials

The following are available online, Figure S1: Response surfaces obtained when using BSA as a precipitant in the sample SKN (A), PIP (B), ELL (C), and GAL (D).; Figure S2: Response surfaces obtained when using PVPP as a precipitant in the sample SKN (A), PIP (B), ELL (C), and GAL (D).

## Abbreviations

Polyvinylpolypyrrolidone (PVPP); Adams–Harbertson assay (AH); Folin–Ciocalteu assay (FC); Methylcellulose precipitable tannin assay (MCP); Optical density 280 nm (OD 280 nm); Bovine serum albumin (BSA); Patatin (PP); Grape skin tannin (SKN); Grape seed tannin (PIP); Gallotannin (GAL); Ellagitannin (ELL).
