# Supplementary material for: Evaluation of Plant-Based Byproducts as Green Fining Agents for Precision Winemaking
Source: Molecules. 2022 Mar 3;27(5):1671. doi: 10.3390/molecules27051671 (PMC8911674; doi:10.3390/molecules27051671)
Supplement: Supplementary file 1 [file molecules-27-01671-s001.zip › molecules-1585291-supplementary.pdf]

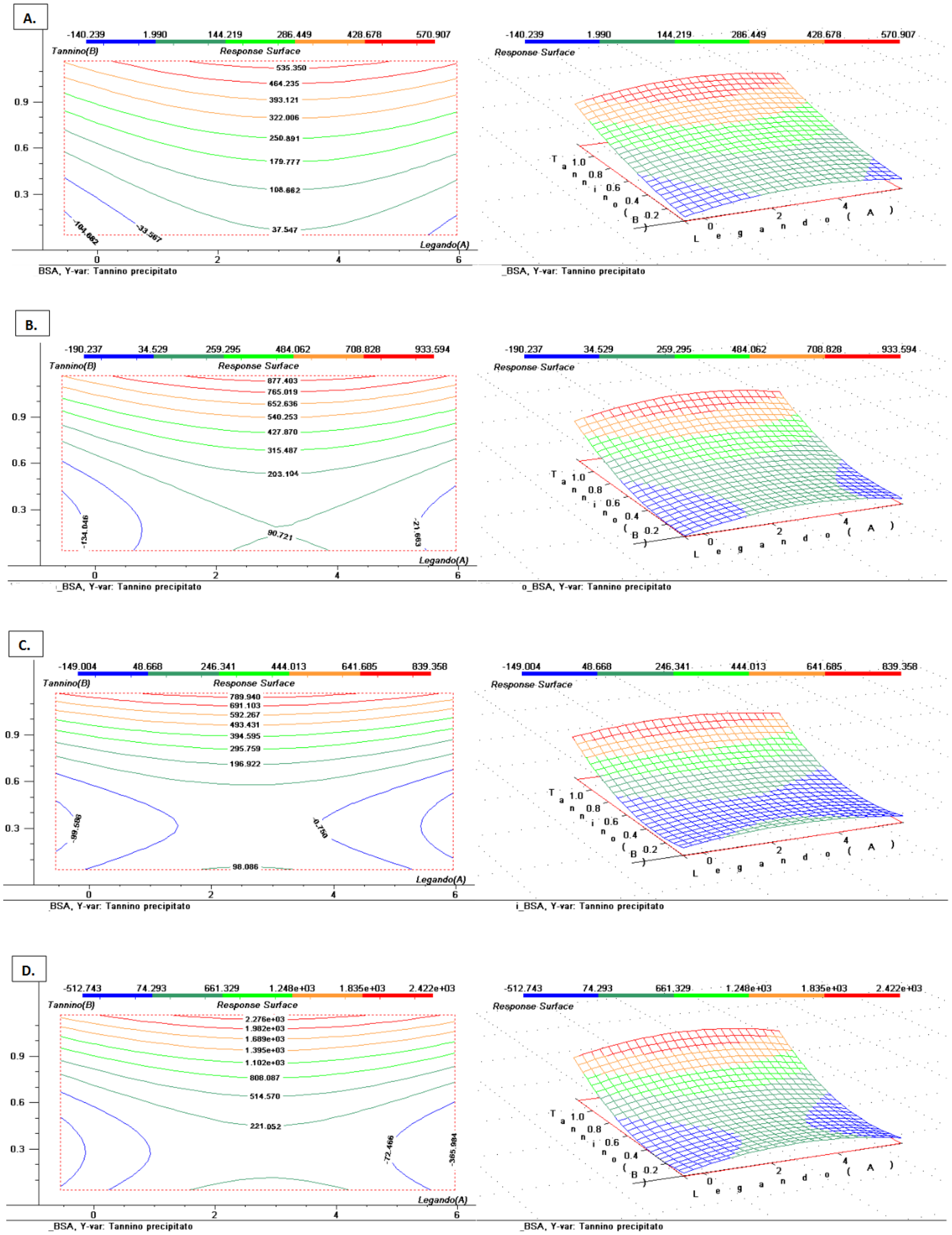


**Figure S1.** Response surfaces obtained when using BSA as a precipitant in the sample SKN (**A**), PIP (**B**), ELL (**C**) and GAL (**D**).

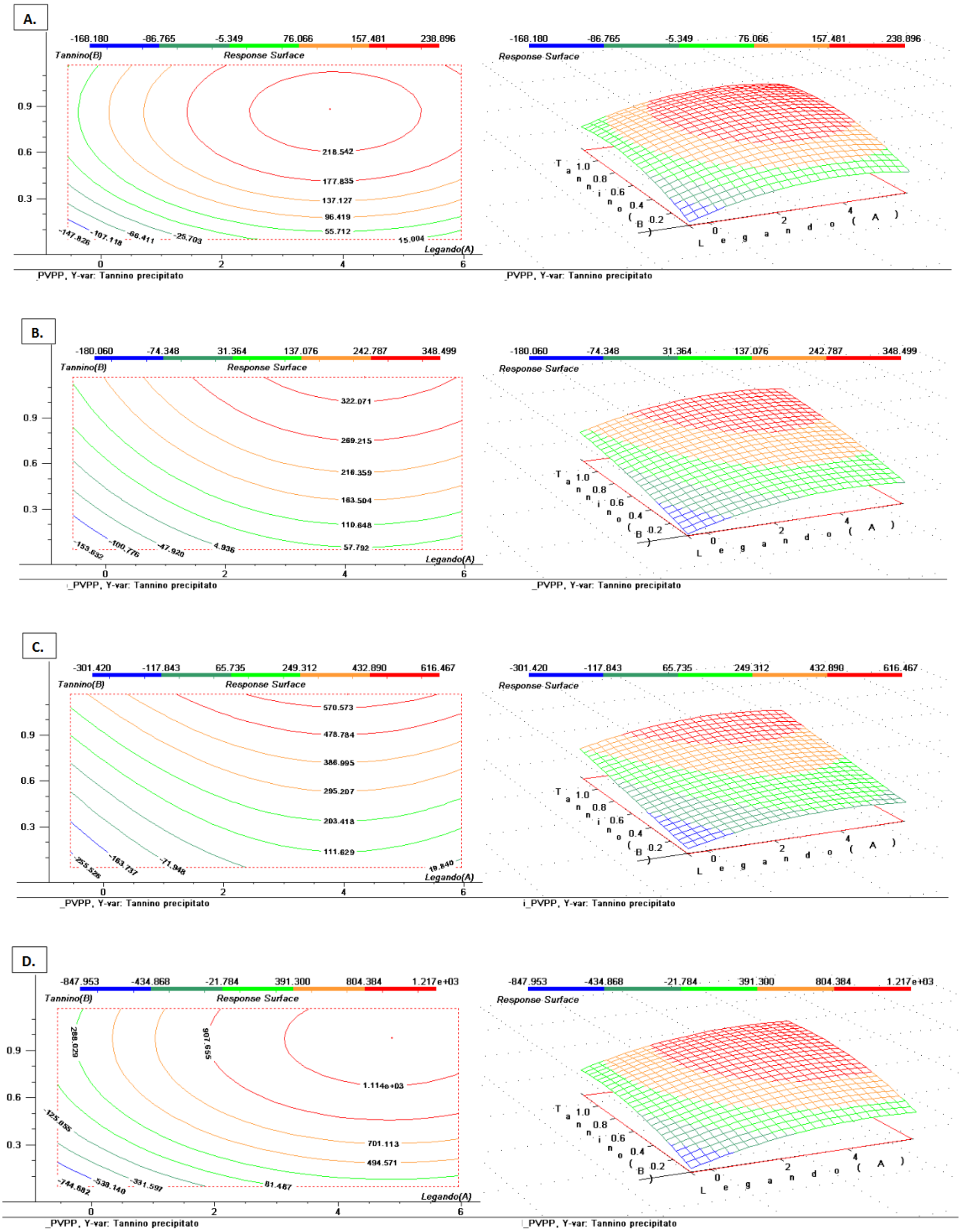


**Figure S2.** Response surfaces obtained when using PVPP as a precipitant in the sample SKN (**A**), PIP (**B**), ELL (**C**) and GAL (**D**).
